# Increased Disarray of Extracellular Matrix Collagen‐I Fiber Network and Compromised Biomechanics in Aortae From Marfan‐Syndrome Mice Assessed Through Combined Opto‐Biomechatronics

**DOI:** 10.1002/advs.77549

**Published:** 2026-09-06

**Authors:** Dominik Schneidereit, Anika Nagel, Vanessa I. T. Zwaans, Laureen Schurr, Jonas Ahr, Michael Haug, Anna Piluso, Alexandra Sporkova, Matthias Karck, Andreas H. Wagner, Oliver Friedrich

**Affiliations:** ^1^ Institute of Medical Biotechnology Department of Chemical and Biological Engineering Friedrich‐Alexander University Erlangen‐Nürnberg Erlangen Germany; ^2^ Institute of Physiology and Pathophysiology Ruprecht‐Karls University Heidelberg Heidelberg Germany; ^3^ Department of Cardiac Surgery University Hospital Heidelberg Heidelberg Germany

**Keywords:** aneurysm, aorta, biomechanics, Marfan syndrome, MechaMorph, mgR/mgR mice, pulse wave velocity (PWV), Second Harmonic Generation, visco‐elasticity

## Abstract

Fibrillin‑1 (*FBN1*) mutations lead to extracellular matrix (ECM) defects with progressive aortic dilation in Marfan's syndrome (MFS). MFS thoracic aortas have increased stiffness, but how visco‐elastic and microstructural abnormalities affect in vivo hemodynamics remains inconclusive. We applied ex vivo uniaxial visco‐elasticity stress‐relaxation testing and simultaneous 3D collagen *Second Harmonic Generation* (SHG) imaging to MFS and wt littermate linearized aortic strips with in vivo vascular function. MFS mouse (*Fbn1* ^mgR/mgR^) aortas were stiffer and more viscous. SHG imaging revealed MFS ECM disorganization at rest and stronger collagen‐alignment strain response than wt strips, indicating greater fibrillar straightening capacity. in vivo, MFS mice developed early, progressive aortic dilation. Pulse wave velocity was elevated in young MFS mice pre‐dilation but declined as aneurysms formed. Female MFS aortic peak pressure and velocity were normal, but reduced in MFS‐males vs. wt. Ex vivo perfused wt carotid arteries were highly compliant. Female MFS carotids were stiffer, had reduced radial strain, a right‑shifted pressure‐strain curve and minimal wall‑thickness changes. MFS carotids did not recover baseline diameter after perfusion, indicating impaired visco‐elastic recoil. We demonstrate early visco‐elastic MFS aortic dysfunction and ECM disorder. Mechanistically, the larger collagen‐straightening capacity in MFS cannot compensate for the increased visco‐elasticity during a beat‐to‐beat cycle.

## Graphical abstract

Combined structure‐function assessment in aortic rings from Marfan mice using *MechaMorph* Opto‐Biomechatronics technology relates increased elasticity (stiffness) and dynamic viscosity and less ordered extracellular matrix 3D‐structure (collagen) as potential causes for impaired Windkessel function and compromised haemodynamics in Marfan's syndrome.

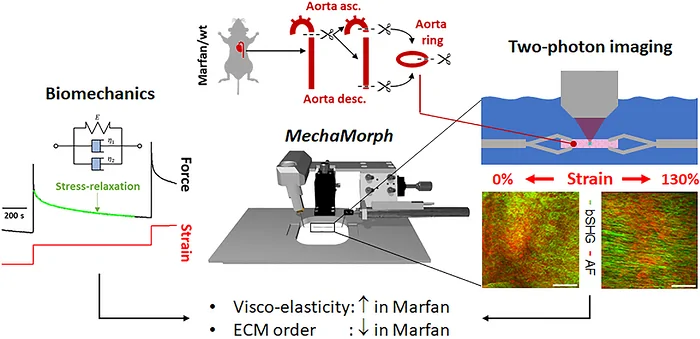

## 1 Introduction

Marfan's syndrome (MFS) is one of the most commonly occurring inherited connective tissue pathologies with an incidence of 1:3,000 to 1:5,000 individuals. There is no preselection for either sex [1], but the clinical course usually is much worse in males [2]. Although predominantly of autosomal dominant inheritance, rare traits of recessive inheritance [3] as well as sporadic mutations in about 25% of cases do occur [1]. Mutations are restricted to *FBN1*, a 65‐exon gene on human chromosome 15q21.1 encoding the connective tissue protein Fibrillin‐1 [4]. Fibrillin‐1 (FBN1) is an extracellular matrix (ECM) glycoprotein with important structural and scaffolding properties for microfibrillar assemblies and long‐term stability of elastic fibers [5]. It also regulates ECM scaffold remodeling through sequestering of latent TGF‐ß binding proteins [6], promotes microfibrillar assembly through N‐terminal domain heparan sulphate binding [7], as well as stabilizing cell‐ECM scaffolding through integrin‐binding of RGD motifs [8]. As FBN1 predominantly associates with insoluble elastin to construct the elastic fiber ECM network during development and remodeling of soft tissues, it markedly guides elastogenesis of, e.g., skin, lungs, eyes, elastic ligaments and large arteries, in particular the aorta [9]. In the vascular tree, disruption of FBN1 expression in transgenic mice has been shown to lead to pathological ageing in arteries, increased aortic stiffness and reduced vasoreactivity, all of which demonstrate the importance of load‐bearing dissipation of vascular strain related to normal FBN1 expression [9]. Biomechanical function in the normal aorta crucially depends on a rather large compliance of its wall, especially in the ascending aorta during the volume storage during systole related to its ‘Windkessel’ function. This is reflected by dynamic interactions among elastin, fibrillin‐1, ECM and smooth muscle cells to accommodate for pulsatile blood flow [10]. A common consequence of increased biomechanical stiffness, fragmentation and loss of elastic fibers as well as increased deposition of extracellular collagen in MFS is, therefore, reflected by progressive weakening of the aortic wall, elastic laminae injuries [11], dissection and/or rupture of aortic aneurysms, all of which seem more severe in males over females, predominantly affecting the ascending aorta and much lesser progression toward the descending or abdominal aorta [12].

The ultrastructural changes and composition of elastin and collagen in the walls of Marfan aortas have long been described by electron microscopy and histology/immune‐histochemistry techniques [13, 14, 15]. However, those require elaborate embedding‐slicing‐staining protocols, may introduce secondary artifacts and are usually restricted to thin slices without 3D information. Multiphoton microscopy (MPM) has become more widely applicable for studying 3D tissue composition and fibrillar arrangement of ECM and intracellular cytoskeletal components susceptible to label‐free non‐linear signal excitation. Such signals may comprise (i) *Second Harmonic Generation* (SHG) for extracellular collagen‐I, intracellular myosin‐II or (ii) two‐photon excited auto‐fluorescence (TPAF) from intracellular molecules, like NADH or FAD, or extracellular elastin [16, 17, 18, 19, 20]. Marfan aorta ultrastructure has been assessed by MPM in excised and (horizontally, transversally) cut aortic ring segments in several studies, confirming significant elastin fiber fragmentation and disorder [19], as well as elastic laminae injuries [11] alongside with increased and disorganized collagen content in the aortic media [15, 21]. However, most studies almost exclusively analyzed microstructure in unloaded excised aortic tissue at idle resting diameter which may very likely deviate from the tunica media organization under in vivo load (i.e., diastolic pressures) and even more so under systolic pressure increases and dynamic wall strain, respectively.

Although several studies have attempted to extract biomechanical data and ultrastructural tissue information in thoracic aorta samples from Marfan mice, both modalities have predominantly been assessed separately [9, 12, 15, 19]. This is because technologies that enable to record biomechanics and ultrastructure through biophotonics simultaneously are scarce, usually custom‐made solutions inherent to single labs and thus, not commercially available. They are often also the result of stringent engineering endeavors in the field of opto‐biomechatronics and tailored to fit a specific application. In terms of spatial design, a biomechatronics solution for simultaneous multiphoton imaging of aorta elastin or collagen components would also have to be designed to keep samples within the working distance of high NA imaging objectives, usually in the sub‐ to lower mm range.

Application of strain to aorta samples ex vivo has been approached in various ways involving intact ring segments with diameter stretch, uniaxial or biaxial wall segment stretch or pressure‐diameter tests using intact long cannulated segments of aortas. Each test regimen comes with pros and cons [22]. Specifically, in order to more closely mimic the multidimensional facet of aortic wall biomechanics in vivo, bi‐ or multi‐axial stretch methodologies are usually preferred over uniaxial ones. However, the formers are usually more cumbersome in design and implementation, while the latter may be more suitable for comparative initial testing. In a series of elegant studies, Cavinato et al. [23, 24] applied an intact cannulated vessel biomechatronics apparatus, developed for biaxial biomechanics testing, consisting of cyclic pressure‐diameter and axial force‐length protocols [22], to intact Marfan mouse thoracic ascending/descending aorta segments (or during normal maturation [24]) alongside simultaneous multiphoton imaging through the intact aortic wall from outside (adventitia out) to assess structure‐function‐relationships [23]. They applied complex cyclic pressure or axial stretch cycles at clamped diastolic‐systolic vascular pressures and given axial stresses, for which it was shown that loaded collagen fiber orientations in the tunica media were primarily circumferentially/diagonally oriented while being primarily axially aligned in the adventitia. However, although graded mechanical loading was applied during the cyclic pressurizations, no information about the in‐process related changes in microstructural fiber re‐ordering was available as images were not taken at increasing loading steps, only at the beginning. Moreover, what is often neglected in biomechanical assessments of vascular and aorta passive material properties is their marked visco‐elasticity in addition to steady‐state elasticity, which is crucial to describe the time‐dependent dynamics of the aorta [25]. Although this can, in principle, be tested using a pressure stress pulse or creep in an environment [23], the vast majority of studies exploit in‐plane stretch cycles, mostly (dynamic) uniaxially, using excised aortic segments [26]. Suitable dynamic testing protocols are: cyclic loading‐unloading and hysteresis analysis, creep tests and stress‐relaxation [27, 28]. To our knowledge, no studies reporting on structure‐function relationships assessing dynamic visco‐elastic material parameters alongside multiphoton assessment of ECM fibrillar reorganization and alignment under mechanical stress exist so far. This requires a simultaneous recording of multiphoton elastin TPAF and collagen SHG at given strain intervals and analysis of fiber orientations.

To investigate the consequences of FBN1 deficiency, we used the mgR/mgR Marfan mouse model carrying a hypomorphic *Fbn1* mutation, which recapitulates key features of the human disease [29]. Male Marfan mice represent a more aggressive Marfan cardiovascular phenotype, with earlier onset, faster progression, and more severe structural deterioration of the aorta. Females show the same underlying FBN1‐dependent pathology but with delayed kinetics and better preservation of aortic integrity, resulting in improved survival. These sex differences reflect both clinical observations in humans and consistent findings in FBN1 mouse models [29, 30]. In Marfan mice, the pathogenesis is characterized not only by structural defects in collagen and elastin but also by broader ECM dysregulation and maladaptive vascular smooth muscle cell responses. FBN1 deficiency promotes a shift of vascular smooth muscle cells toward a synthetic, pro‑remodeling phenotype, accompanied by altered proteoglycan composition and increased activity of matrix‑modifying enzymes [31]. These processes collectively accelerate medial degeneration and contribute to the progressive aortic pathology observed in MFS.

Therefore, we applied our in‐house engineered *MechaMorph* opto‐biomechatronics technology [18], involving uniaxial stress‐relaxation testing alongside simultaneous multiphoton imaging and morphometry analysis to linearized aortic strips from ascending and descending aortas of Marfan and wt littermate mice to extract (i) circumferential steady‐state elasticity moduli (E‐modulus), (ii) dynamic circumferential viscosity and (iii) collagen angular orientations from SHG 3D image volumes from the same sample at various strains, for the first time. We confirm markedly increased stiffness in Marfan aortas, even more so for ascending over descending aorta. In addition, Marfan samples had a much higher dynamic viscosity. From ultrastructural analyses, collagen fiber matrices in the media were much less aligned in Marfan samples but were more ordered in wt mice. As a result of a more straightened geometry in wt aortas, they had a narrower dynamic range for collagen alignment with strain, in particular in the ascending aorta. In contrast, dynamic range was significantly increased in Marfan ascending aorta but similar in descending aortas of MFS and wt mice.

Our ex vivo aorta analyses here are complemented by in vivo data confirming dilated aortic diameters, reduced pressure gradients and blood flow peak velocities in the ascending aorta of MFS mice, being more severe in males over females. We also observe involvement of extra‐aortic vessels in MFS, i.e. the carotid arteries, linked to aortic pathology, as reported previously in human patients [32].

## 2 Materials and Methods

### 2.1 Animals

All animal experiments were approved by the local animal welfare authority (Regional Council Karlsruhe, Germany; approval numbers G‑343/19 and G‑6/25) and conducted in full compliance with Directive 2010/63/EU of the European Parliament on the protection of animals used for scientific purposes as well as current NIH guidelines. Mice were housed in the Heidelberg University Interfaculty Biomedical Facility under standard conditions with a 12‑h light/12‑h dark cycle, with food and water provided ad libitum.The FBN1‑deficient C57BL/6J 129‐*Fbn1^2Rmz^*/J (mgR/mgR, Strain #:005704, RRID:IMSR_JAX:005704, Jackson Laboratories, Bar Harbor, ME, US) Marfan mouse (strain #005704, The Jackson Laboratory, Bar Harbor, ME, USA) represents a gene‑targeted hypomorphic model of Marfan syndrome generated by insertion of a neomycin cassette between exons 18 and 19 of the *Fbn1* gene [5, 33]. The mgR/mgR mice were not maintained as an isolated inbred line but regularly backcrossed to C57BL/6J to minimize genetic drift. Genomic DNA was isolated and genotyped using quantitative real‑time PCR [34].

### 2.2 Aorta Preparation

**FIGURE 1:**
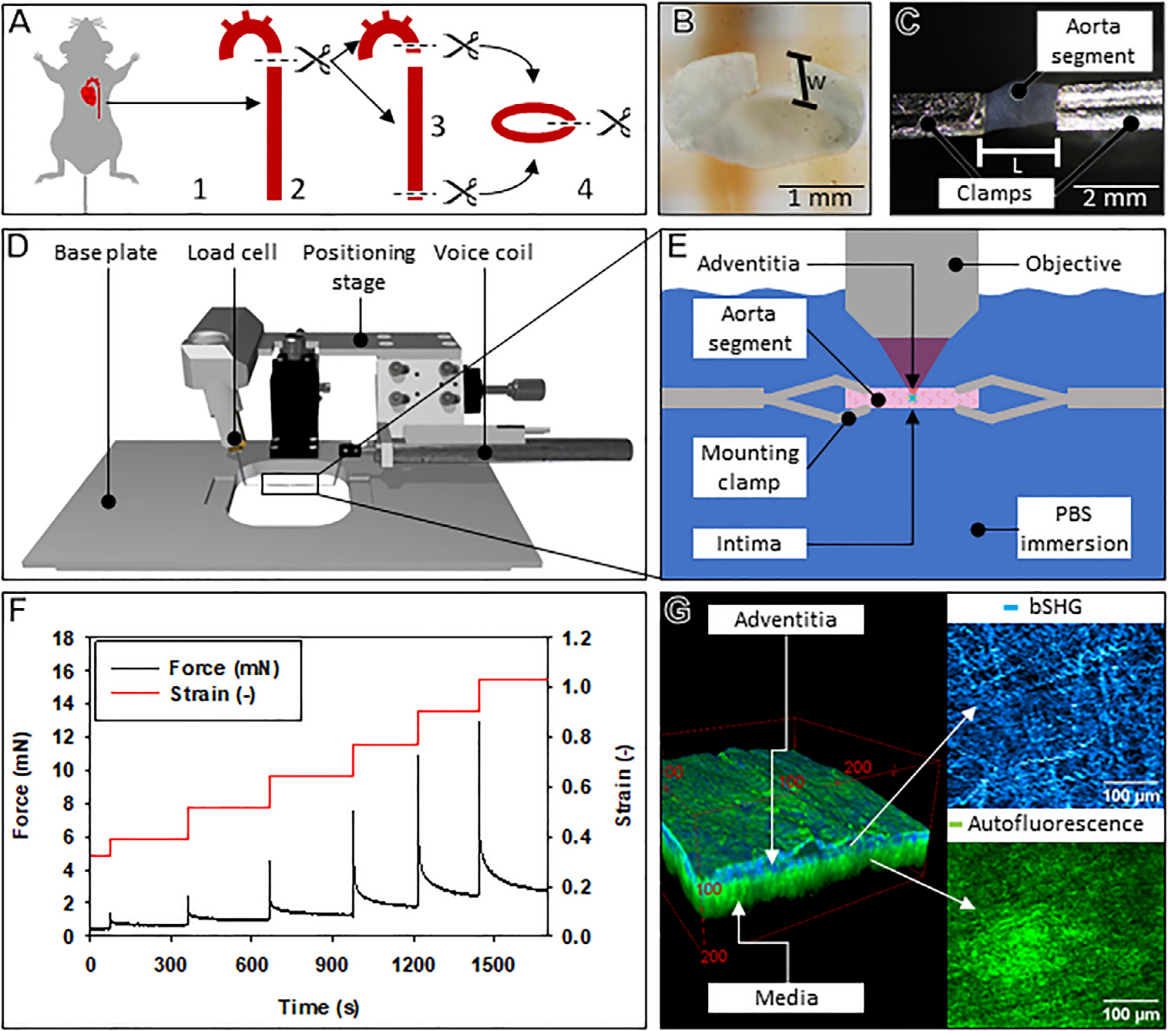
Workflow of experimental procedure: (A) Schematics of aorta segment preparation. (1) The aorta was explanted and segmented (2) into *A. ascendens* (asc.) and *A. descendens* (desc.). Ring segments of 0.5 mm width were sectioned off from asc. and desc. aortas (3). The rings were linearized by one longitudinal cut (4). (B) Reflected light microscopic image of a linearized aorta segment with a width (w) of 0.5 mm suspended in PBS solution. (C) Reflected light microscopic image of a linearized aorta segment that is mounted in the experimental setup with each end affixed to one clamp. (D) The *MechaMorph* opto‐biomechatronics system is shown with its major functional components labeled. (E) A side‐view sketch of the experimental setup shows an aorta segment mounted in the *MechaMorph* in PBS solution and the multiphoton microscope dipping objective optically accessing the segment from above. (F) The force/strain‐time graph shows a typical experimental sequence. Strain on the sample is quickly increased step‐wise by the voice coil actuator, and the passive force response of the sample is recorded by the load cell. (G) A representative 3D reconstruction of an image stack of the aorta sample that was taken during the equilibrium phase of an experimental sequence. The adventitia layer is orientated upward. Collagen fibers are shown in the backscattered bSHG channel (blue, 405/20 nm) while autofluorescence signal (elastin + NADH) is shown in green (525/50 nm).

The aorta samples were provided by the Institute of Physiology and Pathophysiology (Heidelberg University, Germany). The samples were segmented into *A. descendens* (desc) and *A. ascendens* (asc) in phosphate‐buffered saline solution (PBS) and fixed by addition of 4% Paraformaldehyde (PFA) and transferred to the Institute of Medical Biotechnology (Friedrich‐Alexander University Erlangen‐Nürnberg, Germany). There, the samples were further segmented by sectioning off rings of 0.5 mm width from the main trunk (Figure 1A). The aortic rings were linearized by a longitudinal cut (Figure 1B) before being mounted into the *MechaMorph* system with the adventitia facing the objective (Figure 1C).

### 2.3 *MechaMorph* System

The *MechaMorph* system [18] was modified to accommodate the mouse aorta strips for mechanical measurements (Figure 1D). A 100 g TAL221 load cell (LC, SparkFun Electronics, Niwot, Co, USA) was applied as force measuring device with an HX711 load cell amplifier (SparkFun Electronics) to digitize the force readings. An Arduino UNO microcontroller was used to control the load cell amplifier and handle communication with the *MechaMorph* Software on a PC via USB. The force measurement setup provided force readings up to 1,000 mN with a resolution of 24 bit (0.06 µN) at 80 samples per second. The force measurement setup was calibrated with seven‐point calibration before initial use. The calibration was performed by rotating the setup by 90° (aligning the gravity vector to the pull‐force vector in the system), attaching seven sample weights in the range of 0.1‐100 g to the load cell pin and recording the microcontroller output signal. The conversion factor between signal count and mN was determined by the slope of a linear regression between force of weights and load cell count output. The system baseline measurement noise was ∼0.1 mN. The samples were affixed to the tips of the LC and voice coil (VC) pins using micro‐mounting clamps (TW 0.5/0.8, Myotronic, Heidelberg, Germany).

### 2.4 Multiphoton Microscopy

Multiphoton microscopy was performed on an upright TriMScope II (Lavision Biotech, Bielefeld, Germany) microscope using a femtosecond pulsed titan‐sapphire (Ti:Sa) laser with 80 MHz pulse frequency tuned to 810 nm excitation wavelength and 150 mW laser power. A Leica HC FLUOTAR L 25x dipping objective (NA 0.95) with 2.4 mm working distance was used as excitation and collection objective with PBS as immersion liquid. A λ/4 quarter‐wave plate (500 – 900 nm, B. Halle GmbH, Berlin, Germany) was coupled into the beam path after the back‐aperture of the objective [35] in order to circularly rotate the polarization direction of the excitation laser light and to even out differences in SHG signal intensity due to in‐plane collagen‐I fibril orientations [36]. Detection was performed with two photomultiplier tubes (PMT, H 7422–40 LV 5 M, Hamamatsu Photonics, Japan). Bandpass filters were used as excitation filters, using 405 nm (ET 405/20x, Chroma Technology, USA) for *Second Harmonic Generation* (SHG) and 525 nm (ET 525/50m, Chroma Technology, USA) for two‐photon excited autofluorescence (TPAF). Image stacks ‘en face’ with adventitia ‘facing up’ the dipping objective were acquired with a voxel size of 0.77×0.77×1.0 µm^3^ and a field of view of 395×395 µm^2^. The stack depth was adjusted to the sample thickness between 300 and 500 µm. A representative 3D reconstruction of an image stack with projections of SHG and TPAF channels is shown in Figure 1G.

### 2.5 Experimental Procedure for Isolated Aorta Opto‐Biomechatronics Recordings

Each linearized aorta sample was mounted into the mounting clamps (Figure 1E) immersed in PBS. The distance between the tips of the mounting clamps is defined as the initial clamping length of the sample. The initial clamping length was set to a fixed value between 1 and 2 mm, and recorded by the *MechaMorph* software. The sample was straightened from a slackened start configuration by repeatedly stretching the sample for 10 – 20 µm until a passive restoration force response could be detected by the LC. The clamping length before the first force response is defined as the resting sample length for strain calculations (L_0_ at strain ε = 0), the pull‐force on the sample at this position is defined as 0 mN. The *MechaMorph* system holding an aorta sample was placed on top of the multiphoton microscope, and force recordings were started. A 3D mosaic image of the sample was acquired at its center, perpendicular to the mounting clamps. The experiment was conducted in a series of repetitive steps: (1) the distance between the mounting clamps was quickly increased by a set distance between 100 and 200 µm with a speed of 0.2 m/s, stretching the sample almost instantaneously. (2) The force response of the sample was continuously recorded while holding the stretch for at least 900 s to ensure reaching a steady‐state equilibrium force F^eq^. (3) A 3D volume XYZ image stack at the center of the sample was acquired after equilibrating to the actual strain, at this time the force will have exponentially declined to a plateau due to viscous relaxation. Steps (1) through (3) were repeated until the sample started to mechanically fail (after 12 – 17 cycles). A representative output of raw data from the *MechaMorph* software, showing force and strain recordings of an experiment is shown in Figure 1F.

### 2.6 Opto‐Biomechatronics Data Analysis and Statistics

**FIGURE 2:**
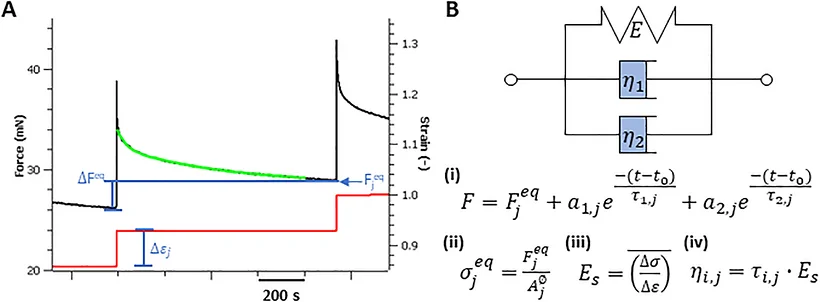
Data fitting and mechanical modelling of passive visco‐elastic aortic tissue biomechanics: (A) The force/strain‐time graph of a single strain step in the experimental sequence is shown with the force traces in black and the strain traces in red. The step‐change in strain Δε results in a force response ΔF(t) with an instantaneous force maximum F_max_ that decays over time and reaches the plateau force ΔF^eq^ in equilibrium. The force decay is described by fitting a second‐order exponential function (B(i)) (shown in green) to the experimental data of each step‐change of strain Δε_j_). The mechanical model (B) of the sample consists of an elasticity component with a Young's Modulus E and two viscous components with dynamic viscosities η_1_ and η_2_. The equilibrium stress σ^eq^ after each strain change j is determined with (B(ii)) using the sample's cross‐sectional area, obtained from the simultaneous microscopy assessment. The Young's modulus of a sample E_s_ is obtained as the slope over all plotted stress‐strain changes in an experimental set on one sample (B(iii)). Each of the viscosities η_i_ is determined for each strain step‐change j using the time constants t_i,j_ (B(iv)).

Using the LabView environment (National Instruments, Austin, Texas, USA) as in [18], both the actuation of the *MechaMorph* VC positioning and the LC voltage output for conversion to force were controlled. The continuous force‐time traces showed the typical behavior of a visco‐elastic material when instantaneously stretched (Figure 2A). Following an instantaneous rise to a maximum peak force F_max_, passive restoration force declined during viscous relaxation to a new steady‐state level in the equilibrium between viscous relaxation and passive elasticity, indicated as F^eq^. As in [18], viscous relaxation could be best fitted with a double‐exponential decay, reflecting a fast and a slow time constant. Using a model of passive visco‐elastic aortic tissue biomechanics, an elasticity element (with a Young modulus E) in parallel with two viscous damping elements η_1_ and η_2_ (dynamic viscosities) was assumed to describe the data (Figure 2B). The time‐dependent force response due to viscous relaxation for a given ‘strain‐jump’ Δε_j_ was described by the equation given in Figure 2B(i), with F_j_^eq^ being the equilibrium force for the same Δε_j_ recording. To obtain the Young modulus E_s_ for each aorta section tested, equilibrium forces F_j_^eq^ at each strain were converted to stress values σ_j_^eq^ using the cross‐sectional area A^∅^_j_ as obtained from the XYZ multiphoton imaging stacks recorded at each Δε_j_ equilibrium (Figure 2B(ii)). E_s_ was then derived from the slope of the respective stress‐strain changes in the range up to 100% strain (Figure 2B(iii)). Dynamic viscosities η were calculated as the product of time constants of viscous decay τ with E_s_ (Figure 2B(iv)). Note that, initially, each dynamic viscosity η_i_ was considered as an independent variable η_I,j_ for each strain recording Δε_j_ which, after testing for Pearson correlation, could then be merged into single η_1/2_ values for each sample s (see Results).

For analyzing the collagen‐I fiber orientation at the equilibrium of a given strain level Δε_j_, the backscattered SHG images (bSHG) from the recorded XYZ stack were processed using Image J and FIJI (NIH software, https://imagej.nih.gov/ij/, https://imagej.net/software/fiji/downloads). Unlike for myosin‐II, SHG signals vanish for collagen‐I fibrils that are oriented in parallel to the laser propagation direction [37, 38]. Thus, only the collagen‐I fibril orientation within the image plane of each XY image was considered relevant for analysis. In linearly polarized laser‐excited images, the SHG signal intensity strongly varies with the orientation of collagen‐I fibrils relative to the laser polarization [39]. By introducing a quarter‐wave plate in the excitation path, this effect was evened out by the resulting circularly polarized laser beam [35]. To analyze the orientation of all collagen fibrils within each XY slice contributing to the XYZ volume, the XYZ stacks were first intensity‐projected by using the FIJI function ‘Z‐projection – max intensity’ which returns the maximum intensity value through the Z‐stack for each XY‐pixel. The XY orientation distribution of the collagen fibrils in the maximum projection image was determined using the plugin ‘Orientation J Distribution’ with a cubic spline interpolation (σ = 2). The local orientation is pixel‐wise calculated using the gradient structure tensor (partial spatial derivatives with respect to the image's fibrils main direction). The local direction within a region of interest is represented by the direction of the largest Eigenvector of the tensor. The orientations were counted and graphically plotted in an angular distribution plot that was fitted with a Gaussian (see Figure 5C). The orientation deviation Ψ was defined as the Gaussian standard deviation and represents the alignment of the collagen fibril cohorts. The smaller Ψ is, the narrower the distribution and thus, the more parallel aligned the collagen fibrils are.

We refrained from quantitative morphometry analysis of elastin fibers due to the strongly overlapping autofluorescence spectra of elastin and NADH [40], for which elastin signals would always contain ‘contaminations’ from cellular sources within the vicinity of the signal origin at the given 525/50 nm emission filter used. Also, since we imaged adventitia‐to‐intima, unlike, for instance, in Lopez‐Guimet et al. (2017) [11], the strong absorption and scattering, while focusing on the collagen‐I layers, led to noticeable reduction in resolution from the elastic fiber layer.

### 2.7 Doppler and B‑Mode Evaluation of Ascending Aortic Diameter, Pulse Wave Velocity (PWV) and Trans‐valvular Gradient

The diameter of the ascending aorta of female and male mgR/mgR and littermate wt mice was evaluated using ultrasound (VisualSonics Vevo 2100 with Imaging Station and a 40‐MHz transducer; Fujifilm VisualSonics, Amsterdam, The Netherlands) at the ages of 4 and 12 weeks. Mice were narcotized with 3% isoflurane and 600 mL/min O_2_ in a specialized chamber. The animals were placed and held in place on an examination surface under continuous volatile anesthesia, and the examination was performed with an MS‐550D transducer (22–55 MHz). The aortic arch was visualized by positioning the transducer in the left parasternal thoracic region to obtain a long‐axis view at a 45° angle relative to the sternum, then rotating 90° for cross‐sectional views using B‐mode and Doppler imaging.

Pulse wave velocity (PWV) was measured in one dimension using pulse‑wave Doppler (PW Doppler). Imaging was adjusted to provide continuous visualization of both the ascending and descending aorta. PWV analyses were performed with the Pulse Wave Velocity Package of Vevo LAB Version 3.1.1. In the PW Doppler recording of the ascending aorta, the interval between the onset of the pulse wave and the corresponding R‑wave of the simultaneously recorded murine ECG was determined. The spatial location of each measured pulse wave was transferred into a longitudinal B‑mode image: the measurement in the ascending aorta provided the arch proximal time, and the measurement in the descending aorta provided the arch distal time. The linear distance between these two time points (the arch distance) was measured freehand on the B‑mode longitudinal image by precisely positioning the endpoints of the drawn line. PWV was calculated by the software as the arch distance divided by the transit time (arch distal time minus arch proximal time) and reported in meters per second (m/s). Although the aortic diameter was measured in both male and female animals, PWV could not be determined in all animals, due to technical problems, or insufficient signal quality. Therefore, the PWV values for both sexes were combined in a single figure (Figure 6C).

Blood flow in the ascending aorta was recorded by Doppler ultrasound to determine the pressure gradient and the peak (maximum) velocity of the blood flow. Peak velocity v_max_ was measured from the Doppler spectral envelope above the aortic valve and reported in m/s. The instantaneous pressure gradient across the measured segment was calculated from the peak velocity using the simplified Bernoulli equation: ΔP = 4 v_max_^2^, and reported in mmHg. Care was taken to align the Doppler beam as parallel as possible to the flow to minimize angle error and to record a stable cardiac cycle for averaging.

### 2.8 Non‐invasive Blood Pressure Measurements

Non‐invasive blood pressure was assessed using tail‐cuff plethysmography (CODA, version 2.1, Kent Scientific, Torrington, Connecticut). Male mice were studied at 10 weeks of age due to premature mortality in this model [29], whereas female mice were studied at 24 weeks. After a 2‐day acclimatization period during which the mice were trained, 25 cycles were recorded per session, of which the first five were excluded [41]. Systolic and diastolic pressures were calculated as the mean of at least 10 valid cycles per animal.

### 2.9 Pressure‑Perfused Carotid Artery Preparation and Functional Assessment

Female MFS mice (22.4 ± 2.0 weeks, 25.9 ± 1.0 g total body weight) and female littermate control mice (age 20.9 ± 2.0 weeks, 25.0 ± 1.6 g total body weight) were anesthetized with isoflurane inhalation followed by cervical dislocation. The common carotid arteries were exposed under a dissecting microscope, carefully excised along their entire length to preserve the adventitia, and transferred immediately into physiological salt solution (PSS, in mM: 140 NaCl, 5 KCl, 1.8 CaCl_2_, 1 MgCl_2_, 10 HEPES, 10 glucose, pH 7.4). Surrounding connective tissue and fat were removed with fine forceps and micro‐scissors, taking care not to damage the endothelium. Isolated carotid artery segments of 2 – 3 mm length were cannulated on glass cannulas, tied with nylon sutures and mounted without axial shortening on a DMT 100P pressure myograph system (Danish Myo Technology DMT, Hinnerup, Denmark). No additional trimming or shortening of the vessel length was performed. Vessels were secured without twisting and checked for leaks. Vessels were equilibrated for 30 – 45 min at 37°C while continuously superfused with PSS. After mounting, vessels were equilibrated for 30 – 45 min to stabilize the resting vessel diameter. Pressure‐diameter relationships were obtained by performing pressure‐diameter curves starting at 20 mm Hg and increasing by 20 mm Hg increments to 180 mm Hg. Afterward, we performed the same step‐wise, but in reverse, reducing intraluminal pressure to the initial 20 mm Hg. Changes in intraluminal and outer diameter were continuously recorded using DMT Data Acquisition software.

For additional histological epifluorescence analysis of carotid arteries, the vessels were excised directly from the pressure myograph system at the glass cannulas immediately following the perfusion experiment, embedded in Tissue‑Tek OCT (Sakura Finetek, Netherlands), and snap‑frozen. Cryosections (7 µm) were prepared, and cell nuclei were stained with DAPI according to standard protocols. Mowiol 4–88 (Merck Millipore, Darmstadt, Germany) was used as a mounting medium. Microscopy and visualization were performed using an Axiovert 2000 M microscope (Zeiss, Germany), the Orca‐Flash 4.0 digital CMOS camera C11440‐22CU (Hamamatsu Photonics, France) and the TissueFAXS scanning and image management software (Version 4.2.6145.1026; TissueGnostics, Austria). Elastin was visualized by collecting autofluorescence signals in the 480 nm (Cy2/FITC) channel (excitation wavelength 488 nm).

### 2.10 Statistical Analysis

For statistical analysis of the ex vivo data, one‐way ANOVA analysis (Sigma Plot, Systat Software) was applied to compare measured data from the two genotypes, wt and MFS, with a *post‐hoc* Bonferroni test (equal variance) or *post‐hoc* Tukey test (no equal variance), where indicated. A p value < 0.05 was considered significant (*), while p < 0.01 was considered highly significant (**). Normality of data was tested using the Shapiro–Wilk test. Data are plotted as box plots (median value: line, quartiles: whiskers 5 – 95 percentiles, minimum/maximum values: x, mean: rectangle) with overlaid individual data points for both sexes, where applicable. To test whether dynamic viscosities (η_1_, η_2_) extracted from each individual strain step Δεj within one sample were dependent on the strain steps (ηj ≠ ηk ∀ Δεj ≠ Δεk), a Pearson correlation was performed using the online tool http://www.socscistatistics.com/tests/pearson/Default2.aspx, or SigmaPlot. In case of no strain‐dependence (i.e., a correlation near zero equivalent to a flat slope in the η−Δεj relationships, respectively), the η values were seen as independent experiments for the statistical analyses (i.e., Figure 4).

The in vivo data were analyzed using GraphPad Prism version 10.0.2 (GraphPad Software), and the results are presented as mean ± SD or SEM from individual experiments. The Mann‐Whitney test was used for comparing two samples with non‐normal distributions, and the Welch's t‐test was used for samples with normal distributions, assuming unequal variances. P < 0.05 was considered statistically significant.

### 2.11 Declaration of AI Use

Grammarly for Microsoft Office Version 6.8.263 was used solely for language editing and text refinement under full author supervision.

## 3 Results

### 3.1 Marfan Aortas are Markedly Stiffer and More Viscous Than Counterparts From wt Mice

**FIGURE 3:**
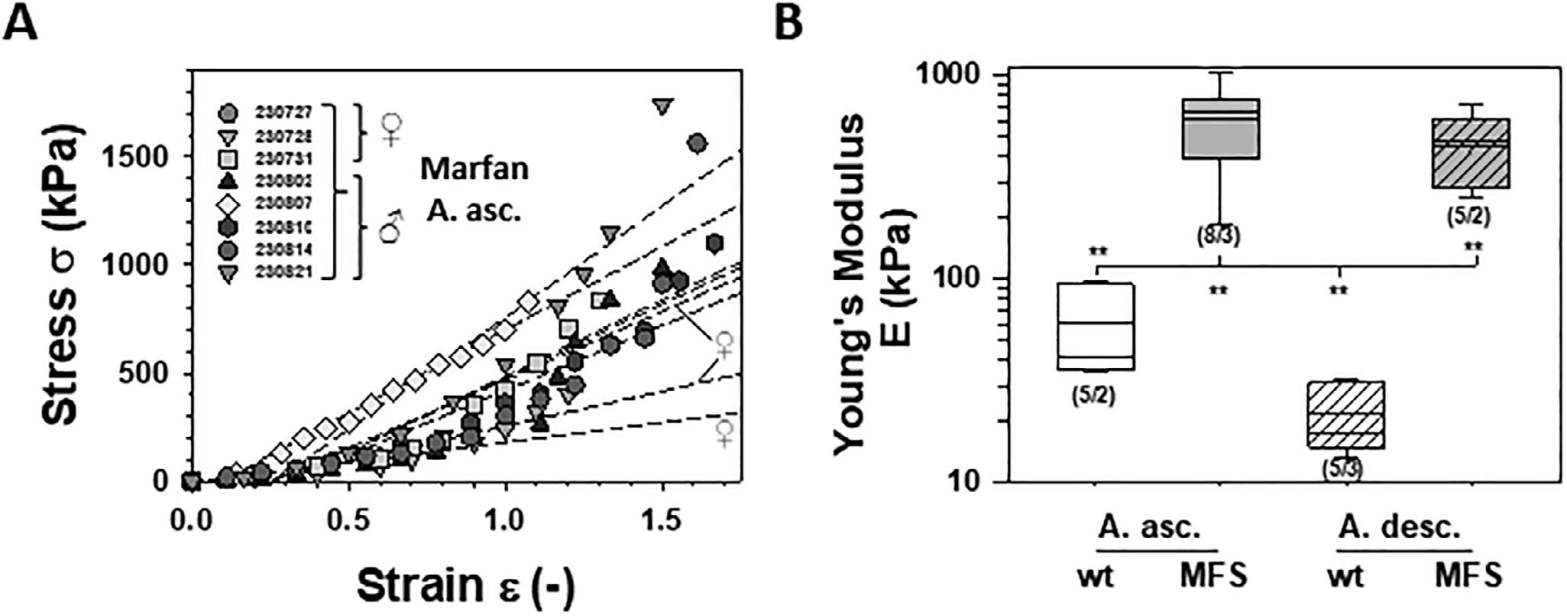
Marfan aortae are at least ten times stiffer than wt aortae. (A) The combined stress‐strain diagram for all samples of the Marfan Syndrome (MFS) *A. asc*. group is shown (each grey scale representing one individual replicate). A linear fit is performed for each sample to determine the mean slope in the linear range (up to e ∼ 1.0) which describes the respective Young's modulus E_s_ of each sample. (B) The E_s_ values from all MFS samples is roughly an order of magnitude higher than the E_s_ values in the wildtype (wt) samples. All differences between sample groups are statistically significant (ANOVA, Tuckey, **: p < 0.001, (m/n) denotes m replicates from n animals). Individual recordings from a female animal are marked in (A).

The instantaneous strain challenges imposed on linearized aorta sections with successively growing amplitudes and maintained passive restoration force recordings allowed to separate both the tissues' elasticity modulus as well as viscous flow behavior as set out in the Methods within the same recording. Figure 3A shows the stress‐strain relationships (extracting σ_j_^eq^ at each strain ‘jump’ Δε_j_) for at least two technical replicates (individual *A. asc*. samples) from the Marfan mice included in the study (in total 8 replicates from one female and two male animals, see Supplemental Table S1). Aortic strips in general showed a linear relationship for strains up to a maximum of 100%, after which the stress started to increase more exponentially. Samples from the one female MFS mouse seemed to show shallower slopes as compared to males, however, this was also subject to variation in the three technical replicates shown (for E‐values, see Supplemental Table S1) and was also not suitable for statistical testing. Therefore, all values were pooled, ignoring potential gender effects at this stage. Figure 3B shows the statistical analysis of the elasticity (Young's) moduli extracted from the linear portions of the stress‐strain curves from all genotypes and samples assessed. From the logarithmic plots, it becomes apparent that aortas from MFS animals, both ascending and descending portions, were highly significantly, at least ten times, stiffer than those from wt animals. Of note, ascending aortas were highly significantly stiffer compared with descending portions in both genotypes.

**FIGURE 4:**
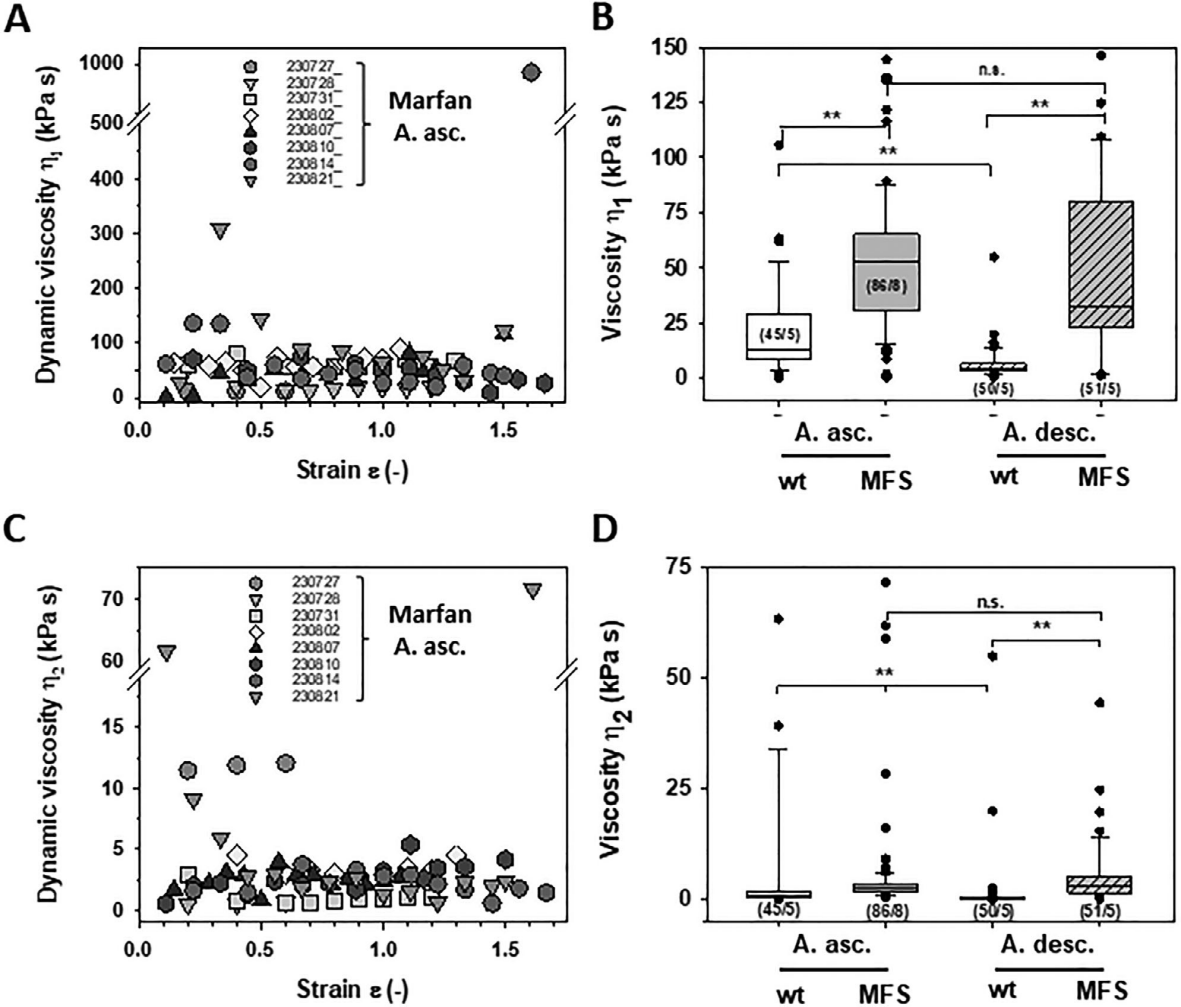
Marfan aorta strips have significantly higher circumferential dynamic viscosities: (A) and (C) show exemplary viscosity‐strain diagrams of all samples in the MFS‐asc. group. Pearson correlation tests show that neither the higher viscosity η_1_ nor the lower viscosity η_2_ are correlated to strain (Pearson p > 0.05). Dynamic viscosities are similar in Marfan asc. and desc. aortas, while ascending aortas are more viscous over descending ones in the wt. Both high (η_1_, (B)) and low (η_2_, (D)) viscosities are significantly higher in Marfan aortas over wt aortas (ANOVA on Ranks with Dunn's, **: p < 0.001). (n/m) in (B) and (D) denotes n individual instantaneous stretch episodes from m technical replicates.

As second component of the visco‐elastic behaviour of the aortic walls' material, their dynamic viscosities were analyzed as given in Figure 2. Figure 4 shows the detailed strain‐dependence of high (Figure 4A) and low (Figure 4C) dynamic viscosities (representing the slow and fast component of restoration force decay during viscous relaxation, respectively) for all MFS ascending aorta replicates and samples. High dynamic viscosities are about ten times larger than the lower ones. A Pearson correlation analysis returned no significant strain‐dependence of viscosities which were then considered as independent measurements and pooled for further analysis as shown in Figure 4B and Figure 4D for η_1_ and η_2_, respectively. A similar picture to the E‐moduli was also revealed for the viscosities, with both high and low viscosities being several‐fold (highly significantly) increased in the MFS samples over the wt samples, both for ascending and descending aorta strips. Within the MFS group, viscosities were not significantly different in both aorta regions, while ascending wt aortas were more (highly significantly) viscous in the ascending over the descending parts.

### 3.2 Extracellular Matrix (ECM) Collagen – higher Disorder at Rest and Larger Straightening Capacity During Consecutive Strains in MFS Aortas

**FIGURE 5:**
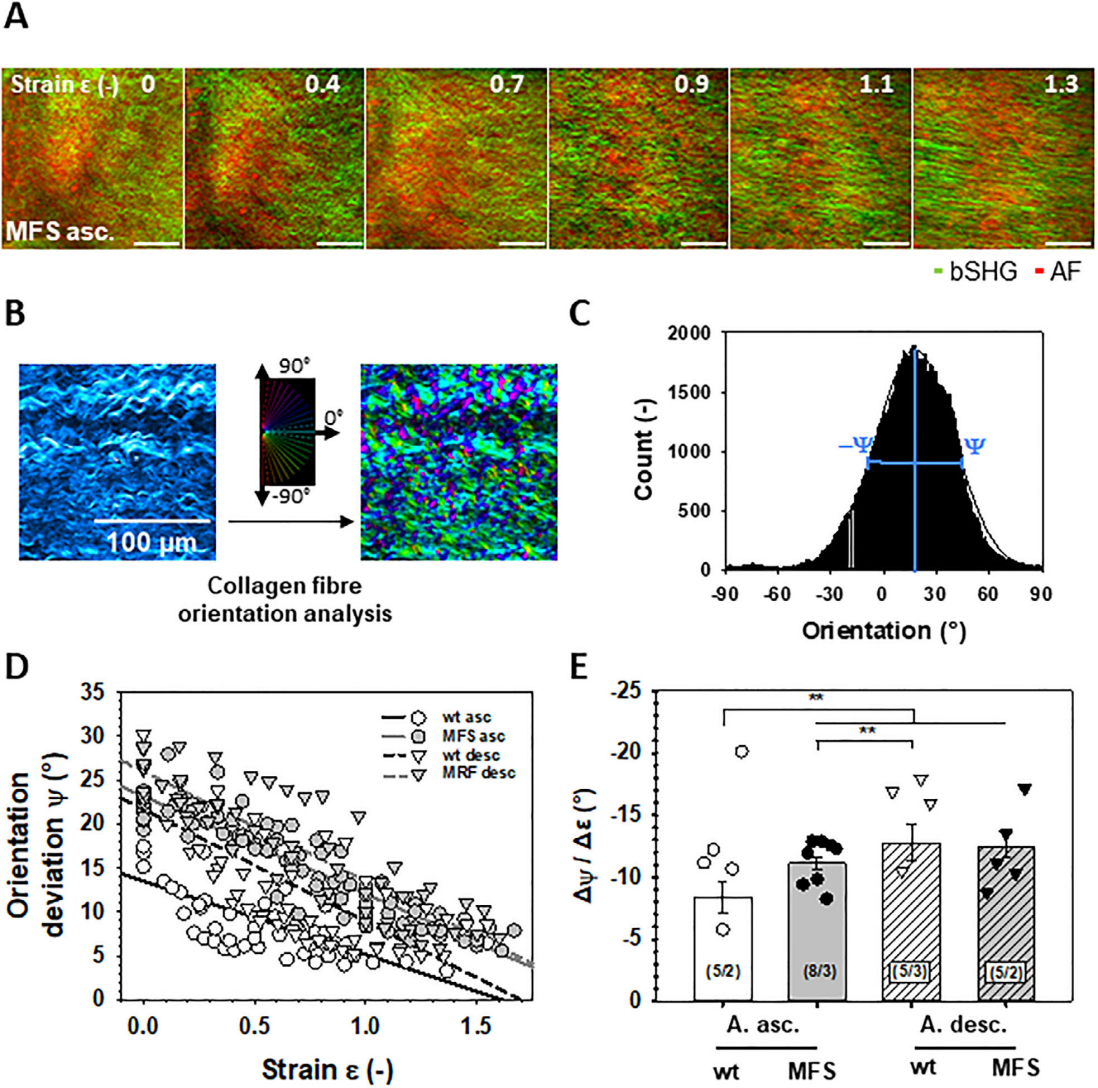
Fiber orientation‐strain correlation: (A) A 3D stack was acquired of each sample after each strain change using simultaneous multiphoton microscopy. Exemplary projections of the stacks at different strain levels of an MFS‐asc sample are shown with the backscattered *Second Harmonic Generation* (bSHG) signal in green and the Autofluorescence (AF) signal in red. Scale bar: 100 µm. (B) The orientations of the structures in the bSHG signal that predominantly originate from fibrillary collagen were determined using the cubic spline method (σ = 2, Orientation J plugin for Image J). (C) The respective orientation deviation Ψ defined as the standard deviation of a Gaussian fit curve over the orientation distribution histogram is shown. (D) The orientation deviation of the collagen fibers exhibits a negative correlation to strain in all samples of all sample groups. The MFS groups exhibit higher orientation deviations (higher disarray of fibers) on all strain levels. (E) Some of the slopes in the Ψ‐ε diagram are significantly different (ANOVA Tukey ** P<0.001, ° P>0.05). The wt‐asc group shows an especially low slope due to already low Ψ (higher order of fibers) at low strains. MFS aortas already start with high Ψ values and thus, have higher alignment reserve under strain as reflected by their slopes. (n/m) in (E) denotes the number of technical replicates (aorta rings) from m animals (see also suppl. Table S1).

MFS aortas have been described to be less ordered in their ECM collagen and elastin spatial distribution patterns with fibrillar breaks and fragmentation in multiphoton images [15, 19]. However, no assessments for the change in ECM architecture during defined successive mechanical stresses are available. As such, it would be important to correlate the increased elasticity observed in MFS aortas in our study (Figure 3) and in the literature to the strain‐dependent repositioning of the ECM lattice. To do so, our previously introduced *MechaMorph* opto‐biomechatronics system exactly allows to delineate this information [18]. For all the mechanical recordings shown in the previous section, the simultaneous SHG imaging of aortic strip XYZ volumes delivered this piece of information. Figure 5A shows projection images from one MFS ascending aorta sample obtained from XYZ image stacks of label‐free autofluorescence (AF, red) and SHG (green) at the holding phase of the indicated strain levels. It is clear that the initial more isotropic and random distribution of fibrillar structures becomes increasingly aligned with the ongoing horizontal stretch. From such intensity projection images, collagen fiber directionality analysis was applied using the cubic spline method in Image J as detailed in the Methods. Figure 5B shows the outcome of such analysis on one example image (left) alongside the colour‐coded angular distribution map (right) from which the orientation distribution histograms were constructed, fitted with a Gaussian fit, and the orientation deviation Ψ being extracted (Figure 5C). The composite analysis of all replicate measurements from the ascending/descending aorta samples from both wt and MFS mice is shown in Figure 5D, plotting Ψ for all tested strains. Three conclusions can be drawn here: (i) the orientation deviations are much higher in MFS over wt aortas, compatible with the higher disarray seen in the Marfan samples (i.e., less aligned and, therefore, broader deviation curve), (ii) ascending aorta wall ECM matrix seems more aligned (lower Ψ values) over descending aortas and (iii), the Ψ‐strain relationship is negative for all samples, indicative of the underlying strain‐induced straightening of collagen fibers and thus, progressive alignment under strain which makes the orientation distribution narrower. Pearson correlation testing revealed a negative linear Ψ‐strain relationship for all individual sample tests, from each of which orientation deviation changes ΔΨ per step strain Δε were derived and plotted in Figure 5E. Note that Figure 5D shows one linear group fit to all the data per genotype and aorta section for visualization purposes only, while in Figure 5E, the slopes from each individual technical replicate were statistically analyzed per genotype and aorta section. Ascending aorta tissue from wt mice showed the smallest ‘strain‐alignment response’ (ΔΨ‐Δε slopes) values, which were significantly higher for descending wt aorta samples as well as for all the MFS samples. Ascending MFS aortic tissue strain alignment was significantly larger over wt tissue, which reflects a higher collagen‐I fibrillar arrangement reserve of those aortas that show much more disarray under unstrained conditions. Interestingly, descending aortas showed similar strain‐alignment of collagen‐I fibrils in both wt and MFS animals although also here, MFS descending aortas showed much higher orientation deviation values at given strain levels as compared to wt descending aortas (Figure 5D) but with similar ΔΨ‐Δε slopes.

### 3.3 In vivo Assessment of Vascular Properties of Wildtype and MFS Aortas

Verifying the visco‐elastic properties measured in isolated aortic segments in vivo is essential to establish physiological relevance and translational confidence. Measurements from excised rings or strips provide controlled, high‐resolution data on tissue mechanics. Still, they inevitably omit key aspects of the native environment: transmural pressure, pulsatile flow, intact endothelial signaling, smooth muscle tone and the coupled interaction between the structural matrix and living cells [42]. These factors modulate stiffness, time‐dependent stress relaxation, and hysteresis, so confirming that *ex vivo* findings persist under more physiological loading and perfusion conditions reduces the risk of erroneous interpretation.

**FIGURE 6:**
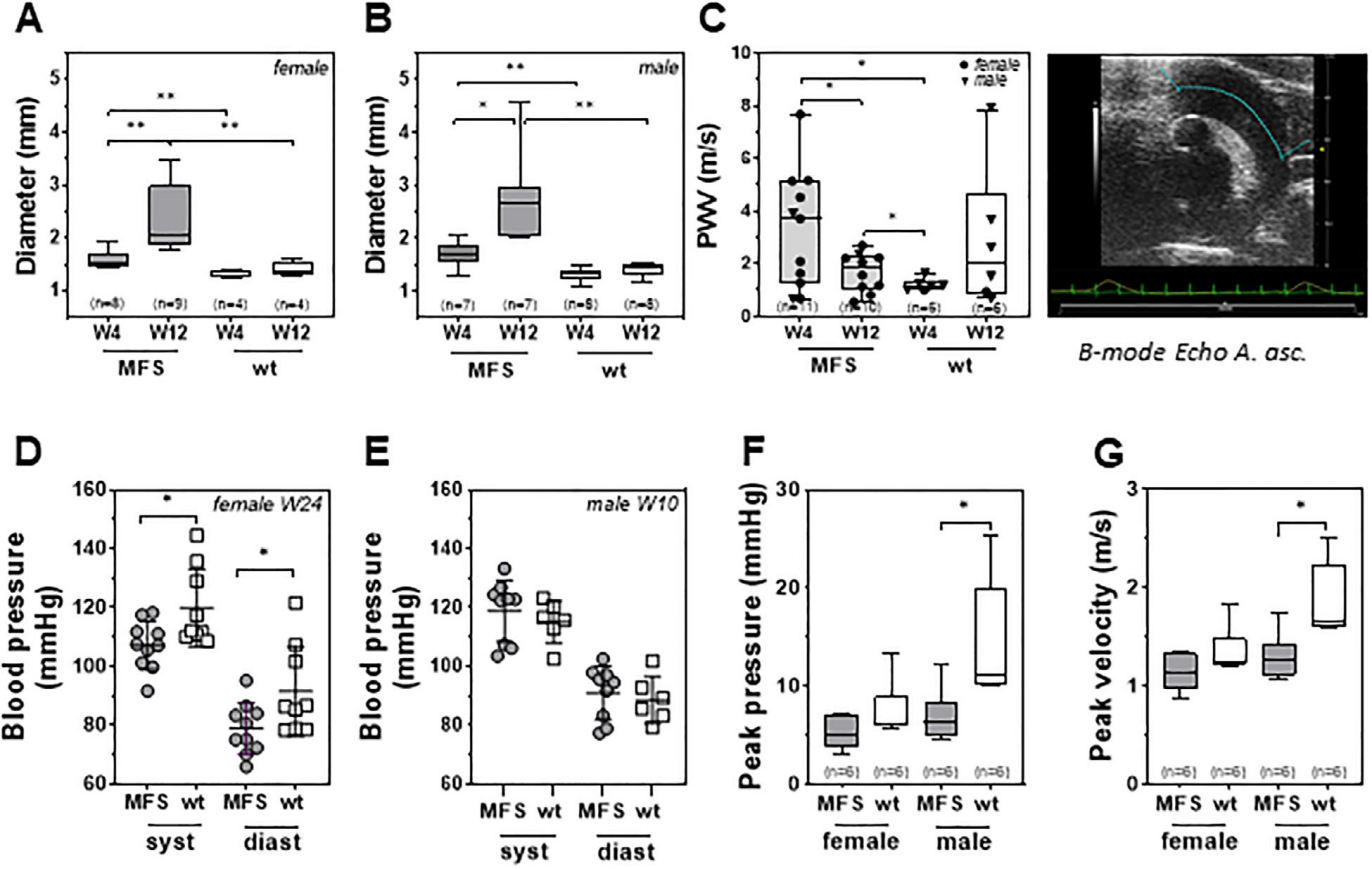
In vivo functional vascular assessment of wildtype (wt) and Marfan (MFS) mouse aorta. Representative imaging and quantitative analysis in live mice using ultrasound and the CODA tail‐cuff system for blood pressure. The data depict dynamic changes in (A, B) ascending aortic diameter, (C) pulse wave velocity (PWV), indicative of arterial stiffness; (D, E) depict systolic and diastolic blood pressure values measured in female and male mice using the CODA tail‐cuff system. The CODA system utilizes volume pressure recording (VPR) technology to noninvasively assess arterial pressure through a dual‐cuff setup placed on the tail. The values for the (F) pressure gradient and the (G) maximum velocity of blood flow in the ascending aorta were determined from ultrasound Doppler examinations throughout the cardiac cycle. The age of the examined animals is given in weeks “W” followed by the number of weeks. The n‐numbers are shown in brackets within the diagrams. The Shapiro‐Wilk test was used to test for normality. The Mann‐Whitney as a non‐parametric test was used for comparing two samples with non‐normal distributions, and the Welch's t‐test for samples with normal distribution assuming unequal variances (*p < 0.05, **p < 0.01).

MFS mice of both sexes exhibited a significant increase in aortic diameter in the ascending aorta associated with aneurysm formation. A measurable divergence from littermate wt control animals was already apparent at 4 weeks and became markedly pronounced by 12 weeks (Figure 6A,B). Although there was no statistically significant difference in overall aneurysm size between males and females, male mice reached relevant critical diameters earlier than females (1.5 – 2.0 mm is considered moderately enlarged, and >2.0 – 2.5 mm is regarded as severely dilated/at risk of rupture [29]). These findings indicate rapid, progressive dilation in this MFS mouse model, with a sex‑dependent difference in the timing of the crucial enlargement despite similar end‑stage diameters. With increasing age, wt mice showed a progressive rise in pulse wave velocity (PWV). In contrast, MFS mice in a mixed‐sex cohort showed a significantly higher PWV already at the earliest time point examined (Figure 6C). As aneurysm formation progressed in MFS mice, reflected by increasing aortic diameter (Figure 6A,B), PWV declined concomitantly, decreasing as the vessel dilated. These findings indicate an early increase in arterial stiffness in the MFS model, preceding macroscopic dilation, followed by a reduction in PWV as the aorta remodels and enlarges.

Female MFS mice at 24 weeks showed a significant reduction in systolic and diastolic blood pressure relative to female littermate controls (Figure 6D). In contrast, male MFS mice at 10 weeks of age, which already exhibit increased aneurysm formation in the ascending aorta compared with female MFS mice, have systemic blood pressure values comparable to those of male control mice (Figure 6E). In the ascending aorta, female Marfan mice showed no difference in peak pressure or peak velocity compared with female wt animals (Figure 6F,G). By contrast, male Marfan mice exhibited significantly lower peak pressure and peak velocity than male wt controls (Figure 6F,G). The hemodynamic values measured in male Marfan mice were similar to those observed in female Marfan and female control mice. Male wt mice had significantly higher peak pressure and peak velocity compared to wt female and MFS males and females (Figure 6F,G).

### 3.4 *Ex Vivo* Carotid Artery Perfusion at Incremental Pressures

Ex vivo perfusion of the mouse carotid artery is often preferable to perfusing a segment of the mouse aorta because the carotid provides a long, straight, surgically accessible vessel with physiological relevance to cerebral circulation, simpler cannulation and pressure control, and lower geometric and branching complexity, all factors that improve reproducibility for graded pressure studies [43].

**FIGURE 7:**
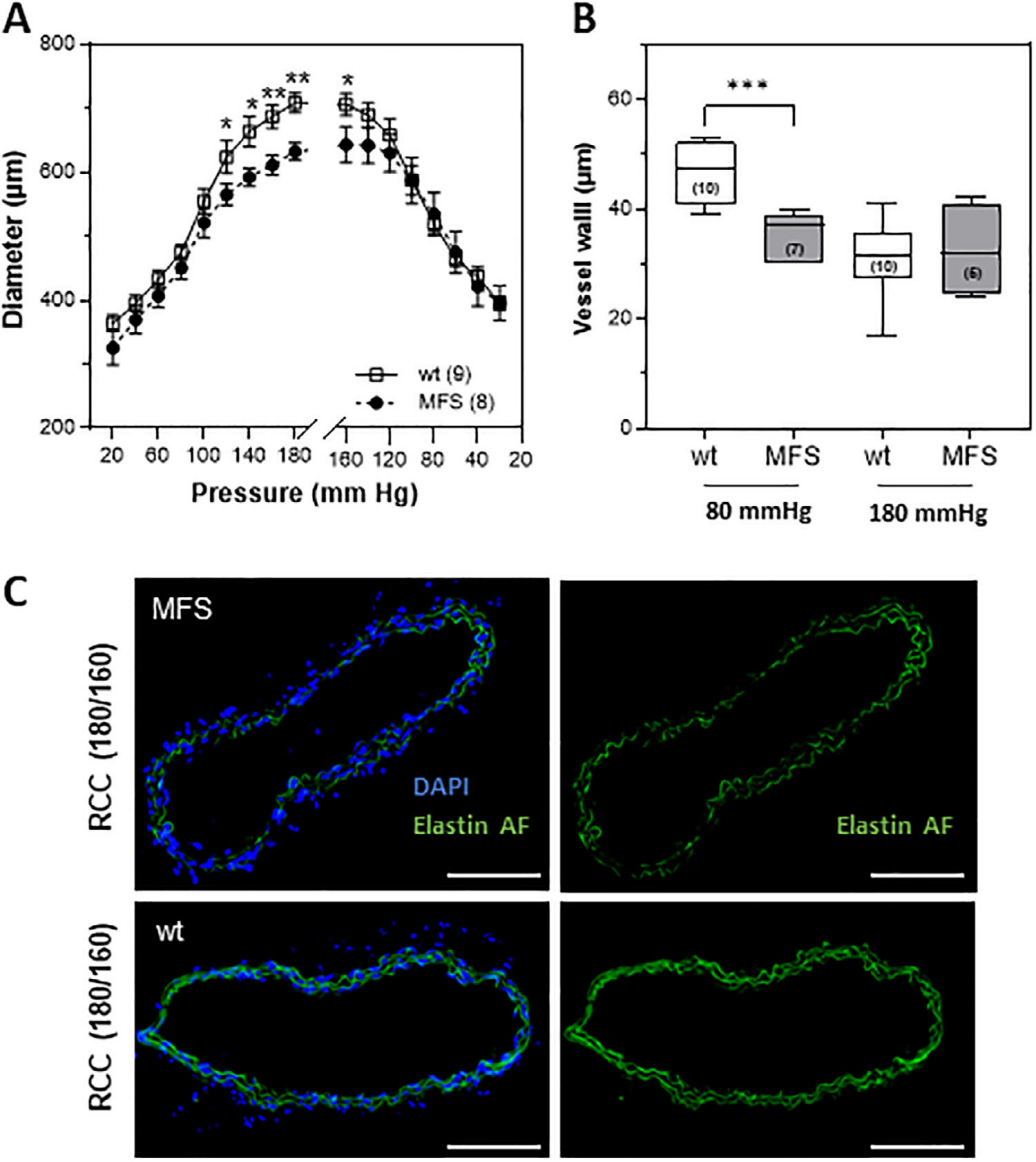
Carotid artery perfusion at graded perfusion pressures. Perfusion experiments were performed on the isolated carotid artery from female MFS mice (22.4 ± 2.0 weeks) and female littermate control mice (20.9 ± 2.0 weeks), while intraluminal pressure was step‐wise increased and decreased (20 – 180 – 20 mmHg). (A) Changes in vascular diameter, demonstrating pressure‐dependent vascular responses. Note that in Marfan mgR/mgR mice (MFS), progressive vascular stiffness impairs the ability of the carotid artery to accommodate pressure increases and fails to fully return to their baseline diameter after pressure perfusion due to viscoelastic hysteresis and irreversible structural changes in the vessel wall as shown in (C) (wt, littermate control mice). Data are presented as mean ± SEM from n  = 8 – 9 experiments (Welch's t‐test). (B) Carotid artery wall thickness at graded perfusion pressures. Wall thickness was measured at a perfusion pressure of 80 mmHg and at a maximal pressure of 180 mmHg. Medial layer destruction led to progressive weakening and focal thinning of the arterial wall in mgR/mgR (MFS) mice but not in littermate control mice (wt). Data are presented as mean ± SEM from n = 5 – 10 measurements (Welch's t‐test). (C) Exemplary epifluorescence images of cryosections from the right common carotid artery (RCC) perfused with an inlet pressure of 180 mmHg and an outlet pressure of 160 mmHg (180/160), immediately following the isolated cannulated perfusion experiments, utilizing the green autofluorescence of elastin. DAPI nuclear staining depicts cells (blue). Scale bar: 100 µm.

Control vessels from female wt littermate mice display relatively high compliance at low pressures (∼80 mmHg), showing pronounced dilation with increasing pressure (Figure 7A). Wall thickness, therefore, decreases markedly from 50 µm at baseline to 30 µm at 180 mmHg (Figure 7B). In contrast, vessels from female Marfan mice are substantially stiffer in this low‑pressure range and exhibit a more minor radial change (Figure 7A), so that wall thickness is already lower at 80 mmHg (38 µm) and shows only a minimal further reduction at 180 mmHg (Figure 7B). These differences produce a steeper pressure‐strain slope for control arteries at low pressures and a blunted, right‑shifted response for Marfan carotid arteries, consistent with reduced distensibility and altered wall mechanics. Note that after perfusion, the carotid arteries of wildtype littermate mice recover their baseline diameter. In contrast, increased stiffness and reduced visco‐elasticity prevented the vessel from the MFS mice from returning to its original diameter (Figure 7A). The medial layer destruction in carotid arteries led to progressive weakening of the arterial wall in MFS mice. Figure 7C shows the disrupted fiber architecture and loss of curvature in perfused MFS carotid arteries.

## 4 Discussion

Mutations in the ECM protein Fibrillin‐1 disrupt ECM scaffolding and elastogenesis and give rise to clinical manifestations of soft tissue pathologies as in Marfan's syndrome [1]. Within that multisystem disorder, substantial fragmentation and decrease in elastic lamellae in the walls of Marfan aortas [44] correlated with increased aortic stiffness and decreased vasoreactivity [45]. Those aspects underline the phenotype of accelerated vascular aging, explaining clinical MFS manifestations such as aortic aneurysms and dissections. Increased circumferential wall stiffness correlates well with aneurysmal propensity and aneurysm formation [46]. However, ‘stiffness’ as elasticity parameter (i.e., Young's modulus) purely reflects a steady‐state material constant obtained from the linear range of stress‐strain curves. Yet, visco‐elastic properties are at least likewise important for vascular function. Especially for the ascending aorta, its profound visco‐elastic compliance is responsible for the ‘Windkessel’ function, i.e., storage of ∼50% of the ejected stroke volume during systole within the dynamically strained aorta to feed continuous flow during diastole. Proximal aortic stiffness thus, impairs this function and is linked to vascular ageing [47], i.e., a pre‐aged vascular phenotype in Marfan's syndrome. As a result, aortic root dilation is the most common cardiovascular manifestation occurring in 60 – 80% of MFS patients [48].

Apart from aortic wall stiffening in MFS, there is growing interest in understanding the underlying micro‐morphological alterations associated with FBN1 deficiency. As such [49], the slope of stress‐stretch curves may encode significant information about ECM architecture, i.e., collagen waviness. This is the rationale for bringing together morphological and biomechanics data to assess structure‐function relationships. For biomechanics assessment, techniques for uniaxial [25, 26], confined biaxial tensile testing of aortic strips [50] as well as biaxial testing of flow‐perfusion cannulated explanted aorta segments [24, 25, 51] have been reported. Those allow to extract both steady‐state elasticity as well as visco‐elasticity using different protocols [27].

Since the elastic region of stress‐strain curves also points toward structural abnormalities within the ECM [49], many previous studies have sought to quantify the ultrastructural disorder in MFS aorta using multiphoton‐excited *Second Harmonic Generation* (SHG) microscopy in addition to elastin fiber networks represented in two‐photon autofluorescence. Collagen fiber angle distributions throughout a blood vessel wall can provide insight into the mechanical behavior of diseased arteries as a consequence of ECM‐remodeling, label‐free and in 3D [11, 52]. Collagen fibers are 100 – 1,000 times stiffer than elastic fibers [53] and thus, outweigh elastin biomechanics by far, in particular when fibrotic ECM collagen‐I deposition is involved. Most recent studies employing SHG have purely focused on structural part and drew conclusions toward biomechanical implications from there. For instance, Cui et al. (2014) [19] demonstrated less collagen fiber organization in aortae from Marfan mice. Similarly, de Souza et al. (2025) [21] found a disorganized collagen fiber network in heterozygous *Fbn1*‐deficient mgΔ^lpn^ mice, similar to MFS patients [54]. However, since they only assessed ultrastructure in excised and resin‐embedded aortic samples, at unloaded conditions, conclusions of ECM remodeling being a correlate of loss of elasticity must be considered with caution as no biomechanical data were extracted in that study.

### 4.1 *Ex Vivo* Structure‐function Relationships in Ascending/Descending Thoracic Aortae From *Fbn1* ^mgR/mgR^ Mice

Our presented approach closes a marked gap in structure‐function analyses of *Fbn1*‐deficient MFS aortic segments by using our opto‐biomechatronics *MechaMorph* system for combined uniaxial tensile testing and simultaneous assessment of label‐free SHG [18] in both ascending‐descending aortic strips. One very crucial advantage of this approach was that while assessing visco‐elasticity using stress‐relaxation material testing [18, 27], we were able to obtain detailed collagen‐I information in 3D with increasing strain. We also report a larger orientation deviation in aortae from MFS mice (*Fbn1^2Rmz^*/J, mgR/mgR) (Figure 5) and a markedly larger biomechanical stiffness (Figure 3), both assessed in the same preparation, for the first time. We are only aware of one study establishing a direct correlation between the progression of micro‐structural defects and associated mechanical properties, with increased circumferential stiffness and reduced elastic energy storage, indicative of increased visco‐elasticity, as shown here by us. In an elegant study using the same hypomorphic mouse model (*Fbn1* ^mgR/mgR^, expressing about 15% of normal fibrillin‐1), Cavinato et al. (2021) [23] applied biaxial mechanical physiological loading to excised and perfusion‐clamped ascending/descending aortas while performing two‐photon imaging through the aortic wall of the clamped segment from the outside (adventitia to intima, as in our study) [24]. They found about 2.5‐fold increased circular stiffness in mgR MFS ascending aorta and ∼1.4 times higher values in descending aortas compared with wt mice, which are much smaller differences than those observed in our uniaxial circumferential stretch approach (about 10‐times higher values in both ascending and descending MFS aortae). Also, their absolute values are about 10 – 15 times higher than ours (lower MPa range as compared with our 20 – 800 kPa range, see Table 1). The comparison of absolute elasticity modulus values is always difficult across studies as those values crucially depend on slope fits to the stress‐strain data in the linear elastic range. Circumferential elasticity obtained from two‐dimensional stress tests was found to match uniaxial tensile stress tests for strains below 50% [25], which is well within physiological aorta strains of 8% – 30% [25]. Most soft tissue samples, including aorta, show exponential augmentation of stress with larger strains [25, 55], which is due to the larger stiffness of collagen fibers [53] starting to prevail once the collagen network has been straightened out (some studies then use tangent moduli to quantify that stiff elasticity part, e.g., [55]). In their porcine aorta biaxial and uniaxial tensile model, Pejcic et al. (2021) [25] describe instantaneous elasticity moduli of 10 – 30 kPa for strains up to 50%, values that are close to our wt E‐modulus values. This is also in agreement with biaxial porcine results from Takada et al. (2023) [50] reporting incremental elasticity moduli of between 330 kPa for strains up to 10% and about 900 kPa for circumferential strains up to 20%. In *Fbn1* ^+/mgΔ^ mice, incremental elasticity moduli of ascending aortas, assessed in excised cannulated aorta segments, were around 0.5 MPa for pressure of 75 – 100 mmHg and somewhat smaller in wt mice [9]. In human MFS patient aortic samples, biomechanics testing yielded differential results. While Jarrahi et al. (2016) [56] found much higher elasticity moduli in ascending thoracic aorta from MFS patients in uniaxial tensile tests (about 7 MPa), other studies reported smaller (low‐)strain or tangent moduli from uniaxial [55] and biaxial planar tensile testing [55, 57] in MFS patient ascending aorta segments.

**TABLE 1:** Comparison of opto‐biomechatronics approach and results from this study and that of Cavinato et al. [23], ATA: Ascending thoracic aorta, DTA: Descending thoracic aorta. TPAF: Two‐photon autofluorescence. adv.: Adventitia. med.: Media.

	This study			Cavinato et al. (2021)		
animal model	129‐*Fbn1^tm2Rmz^*/J			Fbn1^C1041G/+^, Fbn1^mgR/mgR^		
samples	linearized ATA/DTA aortic strips of ∼0.5 mm width, cut‐open and clamped between a force transducer and a voice‐coil actuator pin (linear arrangement)			ATA: aortic root to brachiocephalic trunk; DTA: between first and fourth pairs of intercostal branches Aortas secured on glass cannula for perfusion		
mechanical stimulus	linear strain e (0 – ∼2.0); uniaxial stretch in circumferential direction			perfusion pressure (80 – 220 mmHg), at fixed axial length and combined with axial loading; confined uniaxial and biaxial stretch		
optical two‐photon signals and imaging	SHG: 395–415 nm, TPAF: 500–550 nm; imaging ‘en face’ adventitia facing objective			SHG: 390–425 nm, TPAF: 500–550 nm, cell nuclei: >550 nm; imaging through vessel from outside		
circular stiffness (MPa)		ATA	DTA		ATA	DTA
	wt	∼0.06	∼0.02	wt	∼1.0	∼0.8
	mgR	∼0.7	∼0.6	mgR	∼2.4	∼1.2
				C1041G	∼1.5	∼0.9
axial stiffness (MPa)	not available				ATA	DTA
				wt	∼1.4	∼1.0
				mgR	∼0.9	∼1.3
				C1041G	∼1.0	∼1.1
Dynamic viscosities (kPa s)		ATA	DTA	not available		
	wt	η_1_: ∼20 η_2_: ∼ 5	η_1_: ∼10 η_2_: ∼5			
	mgR	η_1_: ∼50 η_2_: ∼10	η_1_: ∼30 η_2_: ∼10			
Orientation deviation SHG collagen fibers (°)	Note: at idle length (e = 0)			Note: at diastolic load (80 mmHg)		
		ATA	DTA		ATA	DTA
	wt	∼15	∼23	wt	∼20 (adv.) ∼60 (med.)	∼10 (adv.) ∼70 (med.)
	mgR	∼24	∼27	mgR		
				C1041G		
Orientation change (°/e)		ATA	DTA	not available		
	wt	∼−8	∼−13			
	mgR	∼−11	∼13			

One limitation we are aware of in our uniaxial testing is the unconfined nature of the strain that results in biaxial thinning of the two other remaining free axes or degrees of freedom, so that absolute elasticity moduli may be underestimated. However, this should not affect the relative differences between wt and MFS aorta samples being subjected to the same systematic error. Other studies applying biaxial testing in both circular and longitudinal directions showed that longitudinal (‘axial’) stiffness was usually smaller [55] or at least comparable to circumferential stiffness [23] and unrelated to disease phenotype.

One major strength of our approach here is the direct assessment of visco‐elastic behavior through stress‐relaxation tests. Unlike ‘hysteresis’‐tests that predominantly target elastic properties (e.g., energy loss during quasi‐static strain‐relaxation cycles [57]), stress‐relaxation directly measures the kinetics of viscous material ‘flow’ through fluid‐like re‐arrangement of ECM elements and thus, energy dissipation. This allows to derive dynamic viscosity values. Our results (Figure 4) show a markedly increased dynamic viscous behavior in mgR MFS samples in circumferential orientation, both for ascending and descending thoracic MFS aorta segments compared with wt segments. We are not aware of any other study that has provided dynamic viscosity values of MFS aortic strips. Of note, higher values of energy loss imply reduced efficiency in transferring energy upon loading the aortic wall [57]. A significantly reduced energy loss in human MFS ascending aorta strips was determined over healthy patient samples using biaxial loading‐unloading cycles [57]. This is in agreement with our direct kinetics analyses in the mgR mice for both ascending and descending aortas. The presence of two distinct time constants in our recordings (fast vs. slow, reflecting low vs. high dynamic viscosities, respectively) is indicative of two underlying ECM components that contribute with their own viscous modulus to the overall dynamic viscosity. It is tempting to speculate that those reflect elastin and collagen fibers, in particular since the ∼two‐fold orders of magnitude difference nicely aligns with the stiffness differences between collagen and elastin fibers [53]. However, this still awaits experimental confirmation. Interestingly, in a study assessing elasticity moduli of collagen and elastin fibers in descending aorta samples from 50 dead human donors, free from disease and aged 27 to 86 years, elastic fibers' elasticity was calculated to ∼0.5 MPa, while for collagen fibers, it was 300 MPa, i.e., two‐ to three order of magnitude larger, which is also reflected by the approx. two‐fold orders of magnitude difference between η_1_ and η_2_ in our study. Regardless of the exact nature of the underlying fibers responsible for the two kinetics components (i.e., elastic vs. collagen, or alternatively, intact vs. fragmented elastic fibers), the markedly increased viscosity in MFS over wt aorta (asc./desc.) points toward an important impairment of energy dissipation in the thoracic aorta during loading, which may be responsible for a hampered *Windkessel* function as well as compromised pulse wave formation in distal parts (see in vivo section below).

In order to obtain a more distinct picture of ECM architecture with a focus on collagen scaffolds, we took advantage of our *MechaMorph* system to simultaneously obtain 3D‐image stacks of collagen fiber arrangements and analyzed them for angular distributions for each strain‐step. Although SHG imaging has been occasionally used in studies of Marfan aortas [11, 15, 19, 21], only one study so far has combined SHG imaging with biomechanics assessment in the same sample, using excised and perfusion‐clamped aortic sections [23]. That work [23] applied label‐free collagen SHG imaging from the outside of the aorta while clamping them at a diastolic pressure baseline of 80 mmHg. In ascending (ATA) and descending (DTA) aortas from wt, *Fbn1* ^C1041G^ (mild Marfan phenotype) and *Fbn1* ^mgR/mgR^ mice (severe Marfan phenotype), medial and adventitial collagen content and fiber angular orientations were analyzed and correlated to inner aortic diameters under diastolic load. Adventitial collagen fiber straightness was significantly higher in the MFS samples over wt, with loaded vessel diameters increasing in the MFS‐ATA (i.e., highly significantly dilated in the mgR model) but being similar in the MFS‐DTA. Analyzing the angular collagen network distributions, loaded collagen fibers were primarily oriented in the axial direction within the adventitia and in circumferential/diagonal direction within the media (see Table 1). There were no obvious differences in the MFS‐ATA adventitia and media and slightly more axial distribution as compared to wt in the MFS‐DTA ([23], their Figure 3). To conclude, that study suggests more straightened out collagen fibers with more diagonal directions in MFS aorta under loaded conditions, however, assessed at that load only (80 mmHg). It cannot be ruled out that due to the aorta dilation in the MFS animals, alongside with their increased circumferential stiffness, their load conditions already represent differential strain diameters and thus, collagen network distributions. To clarify this, a detailed 3D collagen angular distribution at increasing strain levels would be required to assess the change in angular distribution with different loads (i.e., an orientation‐load curve). This is exactly what we provide in our study here. Indeed, we find that MFS aortic strips show much more isotropic collagen fiber networks already under slack conditions than in wt littermates (Figure 5D). With ongoing strain, the distributions narrow, as expected, through strain‐induced fiber straightening. Since MFS samples already start at larger isotropic values, they have more straightening reserve over a larger strain range than the wt samples and even a larger decremental straightening per strain step (Figure 5E). This fact in itself could potentially suggest a compensatory mechanism for the increased stiffness, i.e. Young's moduli, of the MFS aortas to cope with reduced systolic stretch due to higher stiffness. However, since those morphometric analyses reflect the distribution in the steady‐state of each strain‐step (i.e., after 900 s of maintained strain), this effect is overruled by the increased dynamic viscosities of the MFS samples that prevent a timely dissipation of energy to store within the aorta during a normal cardiac cycle (∼1 s or less in mice). Therefore, it is expected that these morphological‐biomechanics constraints negatively impact on the in vivo hemodynamics of the thoracic aorta, as also shown here. Table 1 summarizes the differential findings of Cavinato et al. (2021) [23] against our findings here.

### 4.2 In Vivo Hemodynamics of Cardio‐aortic Function in *Fbn1* ^mgR/mgR^ Mice

To complement the so far reported ex vivo biomechanics and structural implications, testing in pressure‐perfused preparations or living animals also captures the influence of pre‐stress, active contractility, and the biochemical milieu on visco‐elastic behavior [58]. Pressure and flow alter collagen and elastin recruitment, modulate smooth muscle activation, and engage mechano‐transduction pathways that affect short‐ and long‐term mechanical remodeling [59]. Demonstrating concordance between isolated and perfused/in vivo measurements, therefore, strengthens mechanistic claims, clarifies the limits of reductionist models, and supports the relevance of the results for hemodynamic conditions encountered in health and disease [60].

In wt mice, PWV increases with age [61]. In contrast, in MFS mice, PWV is already elevated early on and then decreases as aneurysm formation progresses, i.e., as aortic diameter increases. This finding is consistent with meta‐analytic evidence demonstrating that Marfan patients exhibit elevated PWV and reduced distensibility, as a direct consequence of increased wall stiffness, in particular, before aortic dilation becomes apparent [62]. Thus, biomechanical stiffness measures can be early predictors of aortic dilation [62]. The visco‐elastic properties of the aortic wall, including its dynamic viscosity, are less commonly measured but are increasingly recognized as important. The *ex vivo* results (Discussion above) show that aortas from MFS mice exhibit markedly increased stiffness and viscosity compared with wt controls. Degeneration of elastic fibers in MFS alters the wall's mechanical response, potentially increasing its viscous damping and altering its handling of pulsatile stress [63]. The local reduction in PWV in the later presence of an aneurysm is a direct consequence of altered vessel structure in MFS: increased diameter, reduced wall stiffness, and disrupted wave propagation. Studies using pulse wave imaging (PWI) and specialized MRI techniques have shown that within the localized area of an abdominal aortic aneurysm, local PWV is reduced compared with healthy vessel segments [64], with the significant expansion in the aortic radius at the aneurysm site being a primary factor. According to the Moens‐Korteweg equation that relates PWV to physical properties, a larger diameter reduces the velocity of the pressure wave, as the pulse is dissipated over a larger, initially more compliant volume [65]. The change in normal vessel geometry to a wide aneurysm sac and back disrupts the uniform propagation of the pulse wave, causing localized turbulence and slower transmission across the dilated segment [65].

It has been shown that blood pressure reduction, like that observed in female MFS mice in our study, is a key determinant of the decrease in arterial stiffening, i.e., a larger compliance [66]. In female animals, systolic and diastolic peripheral blood pressures are lower, consistent with the observed decrease in PWV. After four weeks, a minor aneurysm is detectable, and PWV is elevated, indicating early aneurysm development (Figure 6C). By 12 weeks, the aneurysm is pronounced, and PWV decreases due to local changes in blood pressure and flow (Figure 6D). However, in male MFS mice, we observed no change in peripheral blood pressure despite increased aneurysmal formation, and even comparable values to those observed in control wt littermates (Figure 6E).

Our findings are also in agreement with a sex‑dependent difference between systemic arterial pressure and local aortic hemodynamics in the mgR‐MFS mouse model: female mice develop lower systemic systolic and diastolic pressures by 24 weeks, whereas male MFS mice show early, pronounced ascending‑aortic aneurysm formation with preserved systemic pressures but decreased local aortic peak pressure and velocity compared to wt littermates. At first, sex hormones modulate vascular structure and inflammation, such that estrogen confers vaso‐protective, matrix‑preserving effects, while androgens promote adverse remodeling [67]. Experimental estrogen supplementation reduces aortic dilation and rupture in male MFS mice, supporting a hormone‑mediated explanation for sex differences in aneurysm progression and systemic pressure trajectories [68]. Moreover, local aortic biomechanics and stiffness change non‐linear with genotype, age, and sex: Marfan‑associated fibrillin‑1 deficiency drives extracellular matrix degradation (i.e., elastic fiber fragmentation) and altered collagen/elastin balance, producing regional increases in stiffness and heterogeneous pulse‑wave transmission that can lower or raise local instantaneous pressures independently of brachial or tail‑cuff systemic measurements.

Our measurements show higher peak pressure and velocity in young male wt mice. Du Pont et al. [69] characterize sex differences in murine cardiovascular ageing and the sex‐specific role of smooth muscle cells’ mineralocorticoid receptors (MR). In that study, aortic stiffness, measured by pulse wave velocity, and aortic MR mRNA increased from 3 to 12 months of age in males but not until 18 months in females. In contrast, Marquez et al. [70] demonstrate that the same hemodynamic metric (increased aortic peak velocity) can have different biological meanings depending on age and systemic vulnerability. In aged females, it is associated with reduced physiological reserve and slower recovery. This implies that our finding of higher peak velocity in young males does not contradict the manuscript by Marques et al. [70]. It rather highlights that sex differences are dynamic across the lifespan and that age modulates the relationship between local hemodynamics and health outcomes. Biomechanical and imaging studies emphasize that PWV and local stiffness are better predictors of regional dilation than brachial blood pressure alone [62]. However, accelerated arterial ageing in MFS has been suggested, as aortic stiffness was increased in MFS patients, independently of fibrillin‐1 genotype, and associated with ascending aorta diameter [71]. Alterations in central hemodynamics were present even when the aortic diameter was within normal limits [71]. Ultimately, the aneurysm geometry and intraluminal flow patterns critically determine peak velocity and pressure [72].

Linking the in vivo to the *ex vivo* data, the increased PWV values in pre‐aneurysm ascending aortas match and are explained by increased elastic moduli and dynamic viscosities in our MFS *ex vivo* results to conduct a lateral wave faster in vivo in a stiffer and more viscous material.

### 4.3 Limitations of Our Study

Our findings obtained from the hypomorphic fibrillin‑1 mgR/mgR mouse model cannot be directly extrapolated to patients, because this strain carries a neomycin‑cassette‐retaining *Fbn1* modification that produces a hypomorphic loss‑of‑function state [5]. This is not fully representative of human pathogenic FBN1 variants, which are autosomal dominant and encompass more than 3,000 distinct mutations across all 65 exons [73]. Moreover, the requirement for homozygosity and the early lethal phenotype in male mgR/mgR mice [29, 33] limit tissue availability in our study. Consequently, conclusions drawn from this mouse model must be interpreted with caution when considering the complex and variable human disease. Moreover, the structural organization of the aortic wall differs profoundly between mice and humans, reflecting species‑specific hemodynamic demands and evolutionary scaling. Although both species possess an elastic‑type aorta composed of intima, media, and adventitia, the *tunica media* in mice is relatively thin, typically comprising 3 – 5 elastic lamellae [74]. The normal number of elastic laminae is over 60 in the human aorta [75].

PWV measurement in the mgR/mgR Marfan mouse may be unreliable because the murine aortic arch is very small, and transit times are extremely short, so millisecond timing errors produce large relative PWV errors. Additionally, the animals' very high heart rates complicate precise alignment to the ECG R‐wave noise, and the need for freehand distance measurements on B‐mode images further increase spatial and temporal uncertainty, so many recordings were non‐interpretable or insufficient for robust PWV calculation. For these reasons, we ultimately compiled the remaining measurements that we considered reliable into a single figure (Figure 6C).

Another limitation of this study is the use of 10‑week‑old male and 24‑week‑old female mice for non‑invasive blood pressure measurements, which introduces age as a potential confounder for vascular function. This design was chosen deliberately because male MFS mice develop aortic aneurysms and dissections substantially earlier, and they also progress more rapidly than in females. Therefore, measurements were taken when aneurysm development was comparably advanced in both sexes rather than at identical chronological ages. While this approach allowed assessment of the vascular phenotype at equivalent ‘disease ages’, we cannot fully exclude residual effects of biological age on the outcomes. Future studies including age‑matched controls or longitudinal measurements would help to disentangle age‑related from disease‑related vascular changes.

Also, we are aware of the fact that our *ex vivo* methodology with uniaxial stress‐relaxation testing represents an unconfined tensile test that still leaves one more degree of freedom open as compared to confined biaxial tensile testing or clamp‐perfusion tests that map the confined longitudinal/axial stress‐strain response. Although one could have used additional uniaxial tensile tests on longitudinal strips, our focus here was more on circumferential structure‐function relationships, and there, in particular on the collagen‐I SHG, as collagen fibers with their 100 to 1,000 times larger stiffness over elastin fibers dominate the visco‐elastic properties under increasing loads in the parallel coupling of these two tissue constituents [53].

A further limitation is that the *MechaMorph* stiffness data were collected with an imbalanced sex distribution and showed substantial technical variability. This reduces the ability to detect sex‑dependent effects and may influence the apparent magnitude of the genotype difference between WT and mgR/mgR mice. Still, E‐modulus values were clearly several‐fold larger even for the one MFS female animal compared to all wt females.

## 5 Conclusions

In this study, we close the gap from tissue structure‐function relationships in thoracic aorta biomechanics and ECM remodeling with its implications in vivo within the same colony of Marfan's disease mgR/mgR mice. Using combined uniaxial tensile stress‐relaxation testing of linearized aortic strips from ascending and descending thoracic aorta in the circumferential direction, our findings of markedly increased elasticity (i.e., stiffness) and dynamic viscosities in circumferential direction explains a stiffer phenotype with increased pulse wave velocities despite more isotropic collagen fiber distributions and larger straightening reserve upon strain in the MFS genotype. The latter just simply does not have enough time to accommodate for the increased visco‐elasticity on a beat‐to‐beat basis, which explains the hemodynamic consequences seen in Marfan's disease.

## Author Contributions

**Dominik Schneidereit**: conceptualization, methodology, software, data curation, supervision, formal analysis, validation, investigation, visualization, writing – original draft. **Anika Nagel**: methodology, data curation, validation, formal analysis, investigation. **Laureen Schurr**: methodology, data curation, investigation, validation. **Jonas Ahr**: methodology, software, data curation, validation, formal analysis. **Michael Haug**: methodology, formal analysis, supervision, validation. **Anna Piluso**: methodology, data curation, formal analysis, investigation. **Alexandra Sporkova**: methodology, data curation, investigation, validation, formal analysis. **Matthias Karck**: resources, investigation. **Andreas H. Wagner**: conceptualization, methodology, data curation, supervision, funding acquisition, investigation, writing – review and editing, writing – original draft, visualization, project administration, formal analysis, resources. **Oliver Friedrich**: conceptualization, methodology, investigation, writing – review and editing, writing – original draft, funding acquisition, visualization, project administration, resources, supervision.

## Funding

The German Research Foundation supported the work by grants WA 1511/3‐1 to AHW.

## Conflicts of Interest

All authors declare no conflict of interest and have no relationship with the industry to disclose in conjunction with this project.

## Supporting information




**Supporting File**: advs77549‐sup‐0001‐SuppMat.docx.

## Data Availability

The data that support the findings of this study are available from the corresponding author upon reasonable request.
