# Supplementary material for: Increased Disarray of Extracellular Matrix Collagen‐I Fiber Network and Compromised Biomechanics in Aortae From Marfan‐Syndrome Mice Assessed Through Combined Opto‐Biomechatronics
Source: Adv Sci (Weinh). 2026 Sep 6:e77549. Online ahead of print. doi: 10.1002/advs.77549 (PMC13547705; doi:10.1002/advs.77549)
Supplement: Supplementary file 1 [file ADVS-9999-e77549-s001.docx]

**Supplemental Table 1. Overview of animals' biometric data alongside animal IDs, experimental dates and aorta specimens, alongside with elasticity (E) moduli as obtained from the MechaMorph biomechanics analyses.**

| **Genotype** | **Identifier**  **(biological unit)** | **sex** | **age**  **(wks)** | **experiment**  **date**  **(technical replicate)** | **aorta type** | **E (kPa)** | **aneurysms**  **present?** |
| --- | --- | --- | --- | --- | --- | --- | --- |
| **wt** | 1882_4274 | F | 15.5 | 230607 | *asc.* | 90.22 | no |
|  | 1882_4274 | F | 15.5 | 230611 | *asc.* | 41.89 |  |
|  | 1882_4303 | F | 13.5 | 230822 | *asc.* | 35.20 |  |
|  | 1882_4303 | F | 13.5 | 230830 | *asc.* | 37.24 |  |
|  | 1882_4303 | F | 13.5 | 230904 | *asc.* | 98.52 |  |
|  |  |  |  |  |  |  |  |
|  | 1882_4286 | F | 14.5 | 230605 | *desc.* | 17.69 | no |
|  | 1882_4286 | F | 14.5 | 230613 | *desc.* | 32.49 |  |
|  | 1882_4303 | F | 13.5 | 230703 | *desc.* | 29.64 |  |
|  | 1882_4303 | F | 13.5 | 230705 | *desc.* | 13.43 |  |
|  | 1882_4274 | F | 15.5 | 230612 | *desc.* | 16.54 |  |
|  | | | | | | | |
| **MFS** | 1882_4300 | M | 13.0 | 230810 | *asc.* | 704.18 | Yes, *A. asc.*  mid-large |
|  | 1882_4300 | M | 13.0 | 230814 | *asc.* | 685.23 |  |
|  | 1882_4300 | M | 13.0 | 230821 | *asc.* | 1032.50 |  |
|  | 1882_4302 | F | 13.0 | 230727 | *asc.* | 186.43 |  |
|  | 1882_4302 | F | 13.0 | 230728 | *asc.* | 321.80 |  |
|  | 1882_4302 | F | 13.0 | 230731 | *asc.* | 630.28 |  |
|  | 1882_4304 | M | 13.0 | 230802 | *asc.* | 771.46 | Yes, *A. asc.*  with dissection |
|  | 1882_4304 | M | 13.0 | 230807 | *asc.* | 600.06 |  |
|  |  | | | | | | |
|  | 1882_4300 | M | 13.0 | 230614 | *desc.* | 468.90 | no |
|  | 1882_4300 | M | 13.0 | 230619 | *desc.* | 249.02 |  |
|  | 1882_4300 | M | 13.0 | 230629 | *desc.* | 518.74 |  |
|  | 1882_4304 | M | 13.0 | 230712 | *desc.* | 714.09 |  |
|  | 1882_4304 | M | 13.0 | 230726 | *desc.* | 309.93 |  |
